# Successful management of pleural infection with very low dose intrapleural tissue plasminogen activator/deoxyribonuclease regime

**DOI:** 10.1002/rcr2.408

**Published:** 2019-02-13

**Authors:** Jodi Andrea Hart, Arash Badiei, Y C Gary Lee

**Affiliations:** ^1^ Department of Respiratory Medicine Sir Charles Gairdner Hospital Perth Western Australia Australia; ^2^ Pleural Medicine Unit Institute for Respiratory Health Perth Western Australia Australia; ^3^ Centre for Respiratory Health University of Western Australia Perth Western Australia Australia

**Keywords:** Deoxyribonuclease, empyema, fibrinolytic, pleural effusion, pleural infection

## Abstract

Pleural infection managed with intrapleural therapy using a combination of 10 mg of tissue plasminogen activator (tPA) and 5 mg of deoxyribonuclease (DNase) has been shown in randomized and open‐label studies to successfully treat >90% of patients without resorting to surgery. Potential bleeding risks, although low, and costs associated with tPA remain important concerns. No phase I studies exist for intrapleural tPA therapy and the lowest effective dose has not been established. In patients with high bleeding risks, lower doses may present a safer alternative. We report a case of a complex parapneumonic effusion in a patient with coagulopathy that was successfully treated with a very low dose tPA (1 mg) and DNase (5 mg) regime.

## Introduction

Pleural infection is common. Intrapleural therapy with tissue plasminogen activator (tPA; 10 mg) and deoxyribonuclease (DNase; 5 mg) has been shown to successfully treat up to 96% of patients without needing surgery 1. This dosing regimen was empiric and had not been subjected to dose‐escalation assessment. Hence the lowest effective dose is unknown. The bleeding risk and costs associated with tPA remain clinicians’ major concerns. Recent dose de‐escalation studies found a similar success rate using 5 mg of tPA and further studies using 2.5 mg are underway 2.

Finding a lowest effective dose of tPA/DNase is especially beneficial in patients with high bleeding risks (e.g. coagulopathy) who need pleural drainage. We report the successful use of 1 mg tPA with 5 mg DNase in a patient with coagulopathy.

## Case Report

**Figure 1:**
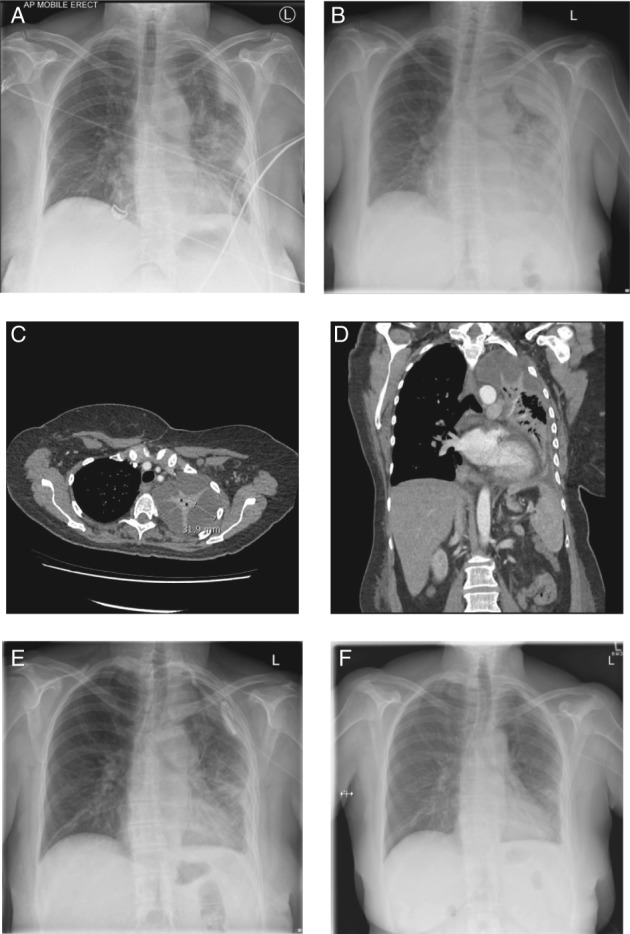
Chest X‐ray and computed tomography (CT) images during treatment. (A) Day 1 – left lower zone consolidation and left upper zone loculated pleural effusion; (B) day 9 – increase in size of left loculated pleural effusion; (C) and (D) day 7 CT – left apical loculated fluid collection; (E) day 12 – persistent loculated pleural effusion with intercostal catheter in situ prior to tissue plasminogen activator (tPA)/deoxyribonuclease (DNase) treatment. (F) Five days after completion of tPA/DNAse treatment.

A 62‐year‐old female presented with a 10‐day history of productive cough, myalgia and fever since returning home from a trip to Sri Lanka. She was febrile (41.1°C), tachypnoeic and hypoxic but haemodynamically stable. Her inflammatory markers were raised (peripheral leucocyte count 19.3 × 10^9^/L and C‐reactive protein (CRP) 420 mg/L). Baseline blood tests revealed deranged liver function (alanine aminotransferase = 379 U/L (normal <35 U/L) and gamma‐glutamyl transferase = 78 U/L (normal <40 U/L)). She was coagulopathic (activated partial thromboplastin time (APTT) of 44.4 s and international normalized ratio (INR) of 1.8). Chest X‐ray (CXR) and computed tomography (CT) of the chest demonstrated left‐sided consolidation complicated by a small loculated pleural effusion (Fig. 1A).

Her past medical history was significant for psoriatic arthritis, which had been managed with methotrexate and sulfasalazine up until six months prior and carcinoma of the breast managed with wide local excision and radiotherapy. She also had a left video‐assisted thoracotomy nine years prior for an undiagnosed effusion which spontaneously resolved after. Pleural biopsies showed benign organizing fibrinous pleuritis only.

She was initially managed with intravenous (i.v.) ceftriaxone 2 g plus azithromycin 500 mg daily. This regime was then changed to i.v. benzylpenicillin 1.8 g 4‐hourly plus clindamycin 600 mg 8‐hourly on day 2 upon identification of *Streptococcus pyogenes* (Group A) in blood cultures. There was initial improvement in symptoms, fever, oxygen saturations, and inflammatory markers (CRP decreased to 36 mg/L by day 5). She was also a subject of a double‐blinded randomized trial and received either i.v. dexamethasone or placebo for 48 h on days 2 and 3. The pleural effusion was not drained because of good clinical response, its small size and the coagulopathy, which persisted despite administration of two doses of i.v. vitamin K 10 mg.

However, she deteriorated and became febrile again at day 6 and her CRP rose to 130 mg/L. Repeat imaging showed a worsening loculated pleural effusion, particularly at the lung apex (Fig 1B–D). Antibiotics were changed to i.v. piperacillin with tazobactam 4.5 g 8‐hourly. An ultrasound‐guided intercostal catheter (ICC) was inserted. Microbial culture of the fluid did not yield any organism.

Over the next 72 h she had minimal improvement. She remained febrile and her CRP remained elevated. Thoracic ultrasound (TUS) and CXR (Fig. 1E) confirmed a persistent septated pleural effusion. Intrapleural instillations with tPA and DNase were commenced. The patient was considered at high risk of iatrogenic haemothorax due to persistent sepsis‐associated coagulopathy (APTT of 53.7 s and INR of 1.4). Hence, a conservative approach using a starting dose of 1 mg tPA was administered initially with a view to dose escalation based on response. The dose of DNase was 5 mg. Intrapleural treatment was given twice daily for 2.5 days (five doses in total).

The patient had significant clinical, radiological, and serological response to the 1 mg tPA/5 mg DNase regime. The volume of pleural fluid drained in the 72 h preceding fibrinolytic treatment was 175 mL and increased to 675 mL in the first 24 h of tPA/DNase treatment and 1450 mL after five doses of tPA/DNase. She was afebrile and her CRP decreased to 49 mg/L. TUS and CXR showed radiological resolution of the pleural effusion and her ICC was subsequently removed (Fig. 1F). Importantly, during the course of treatment she had no bleeding or other complications. At clinical follow‐up two months post‐discharge she remained well with no evident complications.

## Discussion

The optimal dose for intrapleural tPA/DNase therapy remains undefined. Pleural bleeding with 10 mg doses of tPA has been reported at rates between 1.8 and 12% 2, 3. Bleeding risks are dose dependent when tPA is administered intravenously and is likely to be the case for its intrapleural use. In a study of intrapleural tPA use in horses, only a median dose of 3.75 mg (range 0.375–20 mg) was needed 4. This suggests that lower doses may be appropriate and safer. In high‐risks patient using a low dose to start may be a logical approach and may be sufficient. In addition to the reduction of risks, a lower dose therapy will potentially save costs. Currently commercially available tPA comes in vials of various sizes (e.g. 2 mg, 10 mg, and 100 mg) in different countries. This will directly impact the actual cost savings.

In the present case, a patient at a high risk of iatrogenic haemothorax was given a reduced dose of tPA. The successful resolution of her complex parapneumonic effusion, and absence of any adverse effects, suggests that a starting dose of 1 mg of tPA (with 5 mg DNase) administered twice daily for the treatment of pleural infection can be considered in high‐risk subjects.

### Disclosure Statements

Appropriate written informed consent was obtained for publication of this case report and accompanying images. Professor Lee is a National Health & Medical Research Council Career Development Fellow and has received research project grant funding from iCare Dust Disease Authority, Sir Charles Gairdner Research Advisory Committee, Cancer Australia, and the Cancer Council of Western Australia.
